# An Account of Diseases in an Eastern District of London, from the 20th of May, to the 20th of June, 1811

**Published:** 1811-07

**Authors:** 


### *An Account of Diseases in an Eastern District of London, from the 20th of May, to the 20th of June,* 1811.

| ACUTE DISEASES. | |
|---|---|
| Typhus mitior | 5 |
| Febris intermittens | 3 |
| Peripneumonia notha | 4 |
| Apoplexia | 1 |
| Ephemera | 3 |
| Rheumatismus acutus | 5 |
| **CHRONIC DISEASES.** | |
| Tussis | 7 |
| Dyspnœa | 5 |
| Tussis cum Dyspnœa | 9 |
| Raucedo | 1 |
| Hæmoptysis | 3 |
| Phthisis Pulmonalis | 2 |
| Menorrhagia | 4 |
| Leucorrhœa | 7 |
| Amenorrhœa | 3 |
| Diarrhæa | 4 |
| Dysuria | 3 |
| Hæmorrhois | 3 |
| Hypochondriasis | 2 |
| Cephalalgia | 5 |
| Herpes | 4 |
| Psora | 3 |
| Rheumatismus Chronicus | 7 |
| **PUERPERAL DISEASES.** | |
| Menorrhagia Lochialis | 5 |
| Mastodynia | 3 |
| Ephemera | 4 |
| Dolor post partum | 5 |
| **INFANTILE DISEASES.** | |
| Pertussis | 7 |
| Erysipelas Infantile | 3 |
| Tabes Mesenterica | 4 |
| Convulsio | 2 |
| Vermes | 4 |

Fevers of the Typhoid kind still continue to engage the attention of of the medical practitioner. This disease, though it has not, in any of the instances referred to, proved fatal, has still occasioned some perplexity and anxiety. Insidious in its attack, it has hardly been distinguished, till by some more prominent symptoms its character has been discovered. As its approach has been unperceived, so its advances have been slow and gradual; and the progress towards recovery has been unusually tedious.

Amongst children the hooping-cough has been very prevalent. This disease in some instances has appeared in so mild a form, and the parents of the children being accustomed to it, medical assistance was not solicited; but in other cases it has been a more serious and alarming disease, and has too frequently proved fatal. It being of a contagious nature, it is frequently propagated through families where there have been any that were not previously affected by it. It is sometimes at its first appearance mistaken for some other pneumonic affection, in which case there is not a sufficiently early caution to avoid the consequence of a continued intercourse between the patient and others liable to the disease. The cure of this disorder is to be conducted rather by an attention to general diet and regimen, than b y a dependance upon any specific remedy.

